# P02.125. Group acupuncture in an inpatient Joint Replacement Center provides innovative and effective intervention for post-operative pain relief

**DOI:** 10.1186/1472-6882-12-S1-P181

**Published:** 2012-06-12

**Authors:** C Miller, P Weiss-Farnan, B Hopperstad, J Dusek

**Affiliations:** 1 Abbott Northwestern Hospital, Minneapolis, USA

## Purpose

To examine the effects of a group model of acupuncture treatment on pain for joint replacement patients in an acute care setting.

## Methods

In a large midwestern hospital, group physical therapy is provided to joint replacement patients. Orthopedic surgeons added acupuncture to the standard post-operative care for total knee and hip replacements provided through the Joint Replacement Center (JRC). Acupuncture is introduced in the pre-hospital class for surgery preparation and provided immediately after physical therapy on post-operative days one and two. Pre- and post-acupuncture treatment scores for pain are recorded in the electronic medical record. Mean differences in self-reported pain scores were analyzed using a two-tailed t-test.

## Results

Between 1/1/2010 and 9/30/2010, 654 acupuncture treatments were provided to 427 unique patients. The sample included 252 women and 172 men ages 27 to 95 (mean 65.5; SD 12.4 (women) and SD 11.33 (men)). Average pre-treatment pain scores were 4.1, and average post-treatment scores were 2.3, demonstrating a 45.3% decrease in self-reported pain level (p<0.001).

## Conclusion

Group acupuncture significantly decreased self-reported pain in a sample of post-operative joint replacement patients. The addition of acupuncture to the JRC model of delivery is a distinguishing characteristic of the services provided by this midwestern hospital that competes with other hospitals to provide this elective surgery, making it unique among the community options.

